# Lignocellulosic Biomass Waste-Derived Cellulose Nanocrystals and Carbon Nanomaterials: A Review

**DOI:** 10.3390/ijms23084310

**Published:** 2022-04-13

**Authors:** Lindokuhle Precious Magagula, Clinton Michael Masemola, Muhammed As’ad Ballim, Zikhona Nobuntu Tetana, Nosipho Moloto, Ella Cebisa Linganiso

**Affiliations:** 1 Molecular Sciences Institute, School of Chemistry, University of the Witwatersrand, Braamfontein 2050, South Africa; 1477491@students.wits.ac.za (L.P.M.); clinton.masemola1@students.wits.ac.za (C.M.M.); 0708602k@students.wits.ac.za (M.A.B.); zikhona.tetana@wits.ac.za (Z.N.T.); nosipho.moloto@wits.ac.za (N.M.); 2 DSI-NRF Centre of Excellence in Strong Materials, University of the Witwatersrand, Braamfontein 2050, South Africa; 3 Microscopy and Microanalysis Unit, University of the Witwatersrand, Braamfontein 2050, South Africa; 4 Department of Chemistry, Sefako Makgatho Health Science University, Medunsa 0204, South Africa

**Keywords:** cellulose nanocrystals, agricultural waste, lignocellulosic biomass, carbon quantum dots

## Abstract

Rapid population and economic growth, excessive use of fossil fuels, and climate change have contributed to a serious turn towards environmental management and sustainability. The agricultural sector is a big contributor to (lignocellulosic) waste, which accumulates in landfills and ultimately gets burned, polluting the environment. In response to the current climate-change crisis, policymakers and researchers are, respectively, encouraging and seeking ways of creating value-added products from generated waste. Recently, agricultural waste has been regularly appearing in articles communicating the production of a range of carbon and polymeric materials worldwide. The extraction of cellulose nanocrystals (CNCs) and carbon quantum dots (CQDs) from biomass waste partially occupies some of the waste-recycling and management space. Further, the new materials generated from this waste promise to be effective and competitive in emerging markets. This short review summarizes recent work in the area of CNCs and CQDs synthesised from biomass waste. Synthesis methods, properties, and prospective application of these materials are summarized. Current challenges and the benefits of using biomass waste are also discussed.

## 1. Introduction

**Figure 1:**
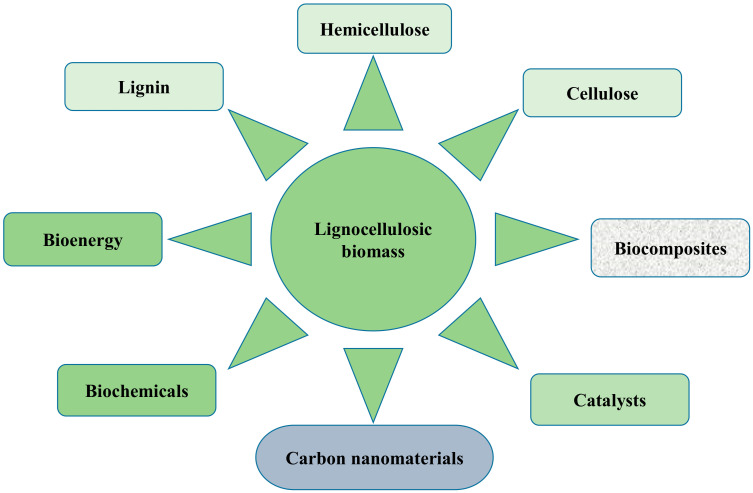
A schematic showing some of the possible extracts from LCB.

The increasing demand for food as a result of population growth has resulted in an increase in agricultural production, which has consequently led to the acceleration of agricultural waste generation. This is accompanied by increased energy demands globally, depletion of fossil fuels, and climate change. Growing research interest has emerged concerning the use of biomass waste material to produce value-added products, due to its potential to form inexpensive and environmentally friendly materials without conflicting with food stock [1,2] Lignocellulosic biomass (LCB) is highly considered as a viable source for renewable energy and an important factor in sustainable economies. The three major building components of LCB are cellulose, hemicellulose, and lignin, with varying percentage composition. These components together with many other products can be extracted as primary or secondary products from LCB, as shown in Figure 1.

Studies on the use of biomass waste for the fabrication of carbon-based materials have emerged recently, such as the use of corncob residue for the fabrication of: porous carbon materials for supercapacitor electrodes [3], hollow spherical carbon materials for supercapacitors [4]), carbon nanosheets for lithium–sulphur batteries [5], carbon nanospheres for use as a high-capacity anode for reversible Li-ion batteries [6], and carbon quantum dots for metal ion detection [7]. Carbon quantum dots (CQDs) are the newest members of the carbon family. Since their discovery in 2004 by Xu et al. [8] and in 2006 by Sun et al. [9], they have gradually become a rising star in the ‘carbon nanomaterials’ family. CQDs are a subclass of zero-dimensional nanoparticles that consist of a carbon core and constitute different functional groups at the surface [10]. They are characterised by quasi-spherical morphology composed mainly of amorphous carbon with sp^2^-hybridised structure and a size less than 10 nm [11]. They exhibit attractive properties such as tuneable photoluminescence, functionalizability, dispersibility, multicolour emission associated with excitation, biocompatibility, size-dependent optical properties, facile synthesis, and low toxicity as compared to their counterparts (semiconductor quantum dots (QDs)) [12]. These extraordinary features make them suitable for potential applications in sensors, catalysis, healthcare, and energy storage devices [13]. In this review, we look into the most recent developments in the extraction of CNCs as well as the fabrication of CQDs from LCB waste.

## 2. Cellulose from Biomass

Cellulose is the most abundant renewable natural biopolymer on Earth. It is a polysaccharide that contains D-glucose units linked together via the β-1,4-glycosidic linkage, and has a general formula of (C_6_H_10_O_5_)_n_, where n is the number of repeated monomeric β-d-glycopyranose units. Cellulose serves as a dominant reinforcing phase in plant cell-wall structures, and its structural details vary depending on the source. Additionally, cellulose is also synthesized by algae, turnicates, and some bacteria [14,15,16]. Naturally occurring cellulose does not occur as isolated molecules, but it is found as assemblies of individual cellulose-chain-forming fibres. These fibrils pack into larger units called microfibrils, which are in turn assembled into fibre. Cellulose has both crystalline (highly ordered) and amorphous (disordered) regions. In the crystalline region, the molecular orientations and hydrogen bonding network vary, giving rise to cellulose polymorphs [17]. Several polymorphs of cellulose exist, namely cellulose I, cellulose II, cellulose III, and cellulose IV [17,18,19,20]. Cellulose I and cellulose II are the most common polymorphs of cellulose. Cellulose I is the native cellulose while cellulose II is obtained via an irreversible mercerization or regeneration of cellulose I [17,20]. The different polymorphs have different properties, such as hydrophilicity, oil/water interface, mechanical properties, thermal stability, and morphology, which contributes to their diverse applications [18,19]. Due to its crystallinity, cellulose I has been used in the synthesis of hydrogels, while cellulose II has been used as a bioethanol feedstock [18]. 

Efficient methods for the isolation of cellulose from LCB such as agricultural waste have recently sparked interest due to the growing interest in developing environmentally friendly and biodegradable materials from waste [21,22]. Cellulose has a wide variety of applications in food, construction materials, paper production, biomaterials, and pharmaceuticals [23]. In recent years, it has attracted a great deal of attention owing to its low cost, biodegradability, high surface-to-volume ratio, good mechanical strength, low environmental impact, abundance, easy functionalization, and versatility in nanoscale processing to form cellulose nanomaterial (nanocellulose) [14,24]. With its diameter in the nanoscale, nanocellulose has drawn a lot of research interest for a variety of applications [25]. Nanocellulose can be further classified into three main groups depending on the size and preparation methods. These three groups are cellulose nanocrystals (CNCs), cellulose nanofibrils (CNFs), and bacterial nanocellulose (BNCs) [26]. Both CNCs and CNFs can originate from LCB, while BNCs can be produced from microorganisms such as Gluconacetobacter xylinus [27]. The nanocellulose field has experienced major developments with reference to its preparation, functionalization and applications in various fields such as nanocomposite membranes, textiles, reinforcing agents, biomedical applications, wood adhesives, adsorbents, and so on [14,25,28]. 

## 3. Cellulose Nanocrystals

**Figure 2:**
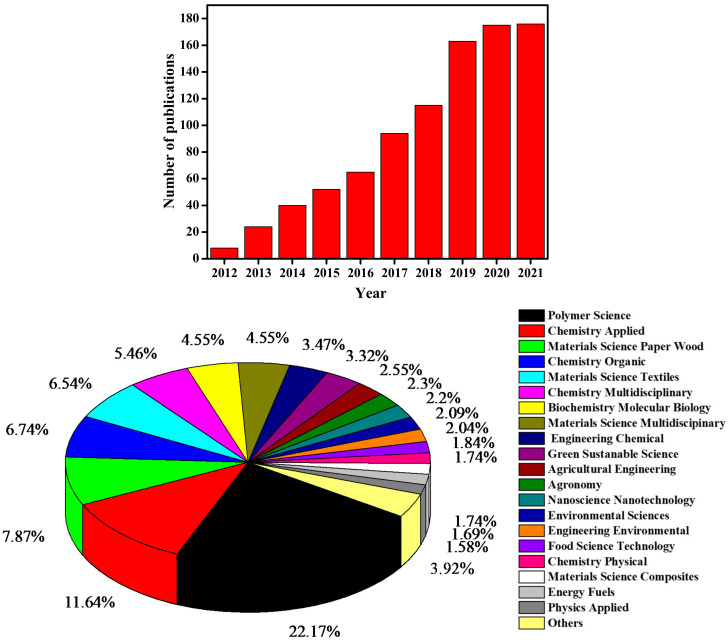
The histogram (top) depicts the number of publications containing “extraction of cellulose nanocrystals” from 2012 to 2021, obtained from the Web of Science in February 2022. The pie chart (bottom) depicts the number of publications (percentage per research field) mentioning “extraction of cellulose nanocrystals” from 2012 to 2021, obtained from the Web of Science in February 2022.

Cellulose has both highly ordered crystalline and amorphous regions in varying proportions, depending on its source. Removing the amorphous region influences the structure and crystallinity of the cellulose, resulting in the formation of CNCs [16,17,29]. CNCs are needle-like particles made up of cellulose chain segments that have been organized in an almost defect-free crystalline structure with at least one dimension less-than-or-equal-to 100 nm [16,30]. CNCs are also known as cellulose nanowhiskers, cellulose whiskers, and nanocrystalline cellulose, but CNCs is the most used term [16,25,30]. CNCs have a high thermal stability, surface area, and crystallinity compared to bulk cellulose, which has more amorphous fractions [31]. Different types of LCB waste have been used to extract CNCs such as cotton [32], pineapple leaf [33], sugarcane bagasse [34], walnut shell [35], soy hulls [30], bamboo fibre [36], and many more. Despite comprehensive research into a variety of biomass wastes, some of the potential natural sources for the development of cellulose nanocrystals, such as corncob, are yet to be widely explored. Figure 2 shows the number of publications with the term “extraction of cellulose nanocrystals” for the past decade (data extracted from Web of Science). More than 160 papers have been published each year for the past three years. The pie chart in Figure 2 also shows diverse fields that find relevance in the extraction of CNCs, although some of the fields overlap (data extracted from Web of Science). 

Various techniques have been employed to prepare CNCs from LCB, which include chemical and mechanical techniques [36]. The two classical chemical treatments are acid hydrolysis and enzymatic hydrolysis, while the mechanical techniques include ultrasonication, high-pressure homogenization, microfluidization, high-speed blending, grinding, and cryocrushing [16,36,37,38,39]. Chemical methods are some of the most commonly used methods for the extraction of CNCs owing to their ease of use, short preparation time, and relatively high yield, whereas mechanical methods require a lot of energy and produce nanocrystal products with a wide range of particle sizes [38,40]. Among the chemical methods, acid hydrolysis is the most common method for the extraction of CNCs [41].

### 3.1. Pre-Treatment of Agricultural Waste

**Figure 3:**
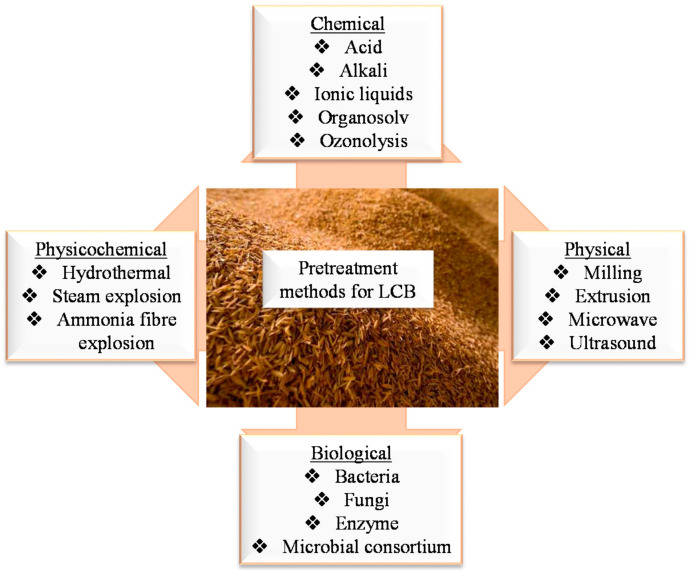
Typical and recent pre-treatment methods for LCB.

LCB does not only consist of cellulose (30–50%), but also hemicellulose (19–45%) and lignin (15–35%) by weight, with the other components including chlorophyll, waxes, ash, and resins [24]. Xu et al. reported that raw corn stover consists of cellulose (44.4 ± 0.4%), hemicellulose (27.8 ± 0.3%), and lignin (19.6 ± 0.2%) [42], while Slavutsky and Bertuzzi reported that sugarcane bagasse consists of cellulose (40.3 ± 1.6%), hemicellulose (21.4 ± 1.6%), and lignin (23.84 ± 0.9%) [43]. Hence, the extraction of CNCs from biomass requires much effort to overcome the crucial pre-treatment stage. It is important to select adequate pre-treatment methods to remove the non-cellulosic material (hemicellulose, lignin, ash, etc.) The recently reported pre-treatment methods for the extraction of CNCs are summarized in Figure 3. These methods are usually selected based on the type of feedstock. For instance, Santos et al. prepared CNCs from pineapple leaves, which contained several non-cellulosic materials [33]. The pre-treatment was conducted with a sodium hydroxide aqueous solution of 2% (*w/w*) to disrupt the hemicellulose and lignin bonds, and a bleaching step with an acetate buffer solution (27 g sodium hydroxide (NaOH) and 75 mL glacial acetic acid, diluted with 1 L of distilled water, and 1.7 wt% sodium chlorite (NaClO_2_) in water) to remove excess non-cellulosic residue. Jiang and Hsieh used two methods to pre-treat tomato peels before the extraction of CNCs [44]. The first method involved the use of acidified-sodium-chlorite delignification, followed by a highly effective alkali treatment using potassium hydroxide (KOH). An alternative chlorine-free route involving alkaline hydrolysis and peroxide bleaching was also developed for comparison using NaOH and 4% hydrogen peroxide (H_2_O_2_). In general, several steps are involved in the pre-treatment stages, including washing and cutting the raw materials into small pieces [45]. To cleave the ester linkages and glycosidic side chains of the lignin, leading to disruption, the source is subjected to alkali pre-treatments at specific conditions. Different alkali solutions have been employed for this process, such as KOH and NaOH [46,47]. This is followed by bleaching (delignification), whereby excess non-cellulosic components are eliminated using sodium chlorite and hydrogen peroxide [31,48]. Extra steps are usually required to dewax the source and clean up the chemical residues [26].

### 3.2. Extraction of CNCs

#### 3.2.1. Acid Hydrolysis

**Figure 4:**
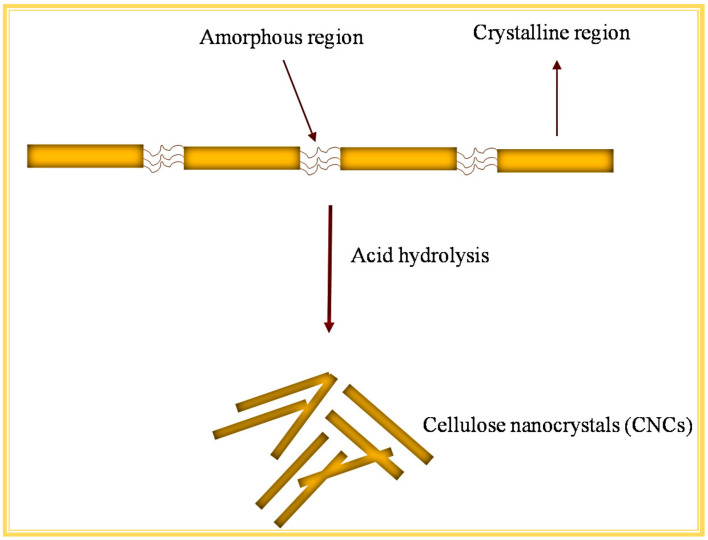
The process of CNC extraction using the conventional acid-hydrolysis method.

The extraction of CNCs from cellulosic fibres usually involves an acid-induced disruption process whereby the glycosidic bonds in the amorphous region are cleaved under controlled-reaction conditions [49] as shown in Figure 4. Various strong acids have been employed to degrade bulk cellulose effectively to release CNCs, such as sulphuric acid (H_2_SO_4_), phosphoric acid (H_3_PO_4_), hydrochloric acid (HCl), nitric acid (HNO_3_), and a mixture of mineral and organic acids [50]. Kassab et al. [51] compared the effects of three different acids on the extraction of CNCs from tomato plant residue (H_2_SO_4_, H_3_PO_4_, and HCOOH/HCl) to form sulphated CNCs (S-CNC), phosphorylated CNCs (P-CNC) and carboxylated CNCs (C-CNC). The produced CNCs exhibited high aspect ratios (up to 98) and high crystallinity (up to 89%), and formed stable suspensions in organic solvents compared to previously reported CNCs from other sources. Wang and colleagues [52] attempted to add phosphate groups to CNCs by phosphoric-acid hydrolysis to improve thermal stability and synthesis conditions. Their results showed that the use of phosphoric-acid medium to obtain CNCs decreased the degradation temperatures; however, thermal stability was still comparable to CNCs obtained from other biomasses that were treated with H_3_PO_4_ and H_2_SO_4_. The acid-hydrolysis treatment with H_2_SO_4_ to prepare CNCs has been widely investigated and appears to be used extensively when compared to other acids. This is because H_2_SO_4_ has been proven to be effective in the elimination of the amorphous components of cellulosic fibres and produces stable CNC suspensions. Figure 4 illustrates the process flow diagram for the extraction of CNCs using conventional acid hydrolysis. As mentioned earlier, depending on the LCB source, cellulose fibres acquired from the pre-treatment stage are then used as a source for CNCs during this step. H_2_SO_4_ hydrolysis introduces sulphate groups to the surface of the extracted CNCs due to the reaction with surface hydroxyl groups of the cellulose through an esterification process, allowing for the formation of anionic sulfate groups [50]. These anionic sulfate groups induce electrostatic repulsion between CNC molecules and promote their dispersion in water [33]. However, the sulfate groups compromise the thermal stability of the CNCs and may contribute to lower yields [33,50]. The thermal stability of the sulfuric-acid-prepared CNCs can be increased by neutralizing the CNCs through dialysis [53]. Overall, the acid-hydrolysis method is simple and can be used to extract CNCs from several agricultural residues. Different agricultural residues that have been used for the extraction of CNCs within the past decade are shown in Table 1. The pre-treatment and extraction processes described above do not differ much; however, the source of cellulose plays a huge role in the dimensions of CNCs as well as related properties and overall yield. While this method is extensively used for CNC extraction, it contributes to high chemical waste disposal, as a result, more strategies for effective, fast, low cost, and environmentally friendly procedures are highly desired.

#### 3.2.2. Oxidation

Oxidation is useful in introducing anionic groups to the cellulose molecules; briefly, it can be separated into two steps. The first step is to oxidize the surface hydroxyl group (-OH) of the pre-treated source and remove the amorphous regions [49]. This results in a structure with negatively charged carboxyl groups (-COOH), which can facilitate the dispersion of CNCs in aqueous solutions and allow further modifications on the surface of CNCs [54]. The most common type of oxidation is TEMPO oxidation. TEMPO (1-oxo-2,2,6,6-tétraméthylpipyridine 1-oxyle) is a stable radical that selectively mediates the oxidation of primary alcohols into carboxylic acids through an aldehyde intermediate [55]. Usually, TEMPO-mediated oxidation is cooperative with mechanical disintegration and selectively oxidizes C6-primary hydroxyl groups of cellulose to sodium C6-carboxylate groups [56]. Zhang et al. [57] used TEMPO oxidation to prepare carboxylated CNCs from sugarcane bagasse pulp with further assistance of ultrasound. Previous studies have used TEMPO-mediated oxidation to prepare carboxylated CNCs; however, this method consists of several steps as well as multiple radical-generating chemicals (sodium hypochlorite (NaClO), sodium bromide (NaBr), and TEMPO reagents), which limit the sustainability of the approach [58]. 

Other oxidation agents such as ammonium persulfate (APS), H_2_O_2_, and nitro-oxidation (using HNO_3_ and NaNO_2_) have also been used to prepare CNCs [59,60,61]. Zhang et al. [61] compared the effects of the preparation methods using TEMPO and acid hydrolysis. Lemon seeds were utilized to extract CNCs by H_2_SO_4_ (S-LSCNC), APS (A-LSCNC), and TEMPO oxidation (T-LSCNC). The results demonstrated that all CNCs maintained cellulose Iβ structure and had a good dispersion regardless of extraction methods, but the T-LSCNC had a higher yield. This is because TEMPO oxidation is also advantageous due to its ability to produce high oxidized yields of up to 90%. Khoshani et al. [62] prepared carboxylated CNCs through one-step catalyst-assisted H_2_O_2_ oxidation. Similar to TEMPO, these two methods require several pre-treatment steps before the extraction of CNCs, while nitro-oxidation decreases the need to consume multiple chemicals, greatly improving the recyclability of the used chemicals [58]. Sharma et al. [59] used one-step nitro-oxidation to prepare carboxylated CNCs from jute fibres, while Chengbo et al. [14] compared the extraction of CNCs using both nitro-oxidation and TEMPO-oxidation from jute fibres. TEMPO oxidation was performed on pre-treated jute, while nitro-oxidation was performed on untreated jute, and both oxidation methods were effective and resulted in carboxylated CNCs with good dispersion and high transparency. The nitro-oxidation extraction process is much less expensive, faster and more environmentally friendly than the acid hydrolysis process. The elimination of the pre-treatment step reduces the amount of chemical waste disposal. This method is also much more sustainable than the TEMPO oxidation process.

#### 3.2.3. Other Methods

Other extraction methods, including but not limited to ionic liquid (ILs) hydrolysis and enzymatic hydrolysis, have been utilized to extract CNCs from agricultural waste [49]. ILs hydrolysis has been highly explored in the field of biomass processing due to its low vapour pressure, low energy consumption, and sustainability [63]. This hydrolysis involves two main steps, the pre-treated cellulose is immersed in an IL for a known period to allow swelling, and water is added to initiate the hydrolysis [49]. During the reaction, the hydrogen and oxygen atoms of amorphous cellulose are easily accessible by the dissociated IL to form the electron donor–electron acceptor. The -OH groups break, leading to the selective removal of the amorphous region [64]. The above-mentioned extraction methods require the use of chemicals, while enzymatic hydrolysis uses cellulolytic enzymes known as cellulases (mixtures of endoglucanases, exoglucanases, and cellobiohydrolases); these are an interesting class of enzymes possessing the ability to act as catalysts for the hydrolysis of the cellulose [65]. These enzymes have specific functionalities that can selectively depolymerize the amorphous region of cellulose to prepare CNCs with high crystallinity [65,66]. This is the most environmentally friendly and low-cost process, as it eliminates the use of toxic chemicals and consumes relatively little energy. However, this process is relatively slow. 

The preparation of CNCs through physical/mechanical processes, such as high-pressure homogenization, grinding, and steam explosion are discussed. During mechanical treatment, pre-treated cellulose pulp is subjected to high shear force, which helps extract the CNCs along the longitudinal direction [67]. High pressure homogenization has been widely employed for the mass production of CNCs in industries due to its simplicity, high efficiency, lack of a requirement for organic solvents, and production of uniform CNCs with high yield [37]. During the homogenization process, pre-treated cellulose pulp is diluted with water and passed through a tiny gap between the homogenizing valve and an impact ring under high pressure (69–207 MPa) and high velocity at room temperature [67,68]. However, high-pressure homogenization involves several passes, which results in high energy consumption [69]. Other mechanical methods include grinding, whereby the cellulose pulp is passed between static and rotating grinding disks, which can be adjusted to reduce clogging [68]. The above-mentioned mechanical methods require mechanical degradation tools; on the other hand, steam explosion facilitates chemical treatments and improves the removal efficiency of non-cellulosic materials through pressure [70]. Steam explosion is a process which consist of heating biomass using saturated steam with high pressure followed by rapid decompression; in this process, the high pressure results in hydrolysis of the glycosidic and hydrogen bonds to produce CNCs [71]. 

**Table 1:** Selected articles showing the extraction of CNCs from different agricultural residues in the past decade.

Agricultural Residue	Pre-Treatment Conditions	Extraction Conditions	CNC Diameter	References
**Mengkuang leaves**	Alkaline treatment using 4% NaOH at 125 °C for 2 h, bleaching using 1.7 *w/v*% NaClO_2_ at pH 4.5 and 125 °C for 4 h.	Acid hydrolysis was carried out using 60 wt% H_2_SO_4_ solution at 45 °C	2–5 nm	[29]
**Mango seed**	Alkali treatment using 2% NaOH at 100 °C for 4 h, bleaching with a solution made up of equal parts (*v:v*) of acetate buffer (27 g of NaOH and 75 mL of glacial acetic acid, diluted to 1 L of distilled water) and aqueous chlorite (1.7 wt% NaClO_2_ in water) at 80 °C for 6 h.	Acid hydrolysis was performed at 40 °C for 10 min using H_2_SO_4_ (11.21 M).	4.59 ± 2.22 nm	[72]
***Agave tequilana* and barley**	The ground fibres were dispersed in an acid solution (0.2 wt% of acetic acid) of 0.27 wt% of NaClO_2_ and 0.7 wt% NaOH kept at 70 °C and stirred for 1.5 h. The sample was then treated with 17.5 wt% NaOH for 30 min.	Acid hydrolysis was performed at 50 °C with 65 wt% of H_2_SO_4_.	MCC 16 ± 6 nm *A. Tequilana* 11 ± 4 nm Barley 10 ± 4 nm	[46]
**Tomato peels**	Tomato peels were placed in oluene/ethanol (2:1, *v/v*) in a Soxhlet apparatus for 20 h to remove wax, phenolics, pigments, and oils. The bleaching was achieved by 1.4% NaClO at pH 3.5 adjusted with acetic acid kept at 70 °C for 5 h, then treated with 5% KOH solution at room temperature for 24 h and then heated at 90 °C for 2 h.	Acid hydrolysis was performed using H_2_SO_4_ (64 wt%) at 45 °C for 30 min.	3.5 ± 5 nm	[44]
**Sugarcane bagasse**	The sugarcane bagasse was pre-treated with ethanol/water (1:1 *v/v*) solution at a solid/liquid ratio of 1:10 at 190 °C for 2 h. The pulped bagasse was then bleached using 24% H_2_O_2_ and 4% NaOH at 70–80 °C for 1 h.	Acid hydrolysis was performed at 50 °C in preheated 65 wt% H_2_SO_4_ for 40 min.	6 ± 1 nm	[73]
**Groundnut shells**	The shells were put under Soxhlet extraction for 8 h using benzene: methanol (2:1 ratio) as solvent. The de-waxed shells were subsequently bleached by treatment at 70 °C for 2 h with 1.5% (*w/v*) NaClO solution at pH 3–4 adjusted by 5% glacial acetic acid. The sample was then treated with 1 M NaOH solution at 65 °C for 2 h.	Acid hydrolysis process using 65 wt% H_2_SO_4_ for 75 min at 45 °C.	9 nm	[74]
**Coffee husk**	Alkali treatment was carried out with a 4 wt% NaOH solution for 3 h, the sample was then bleached using equal parts of acetate buffer solution, NaClO (1.7 wt%), and water for 4 h.	Acid hydrolysis treatment was performed using 64%, wt H_2_SO_4_ at 50 °C for 40 min	20 ± 4 nm	[75]
**Pineapple crown waste**	Alkaline treatment was performed using 5% NaOH solution at 90 °C for 1 h, the sample was then subjected to bleaching using a mixture of 16% (*v/v*) H_2_O_2_ and 5% NaOH at 55 °C for 90 min.	Acid-catalyzed hydrolysis method using 60 wt% H_2_SO_4_ at 45 °C for 1 h.	39 ± 12 nm	[76]
**Cucumber peels**	The sample was pre-treated with 1 M HCl solution for 1 h at 80–85 °C, followed by alkali treatment using 1 M NaOH for 1 h at 80–85 °C. The sample was bleached using 4% (*w/v*) NaOCl for 1 h at 90–95 °C.	Acid-catalyzed hydrolysis method using 60 wt% H_2_SO_4_ at 45 °C for 1 h.	32.9 nm	[45]
**Corncob**	The sample was pre-treated with 1 M HCl solution for 1 h at 80–85 °C. Followed by alkali treatment using 1 M NaOH for 1 h at 80–85 °C. The sample was bleached using 4% (*w/v*) NaOCl for 1 h at 90–95 °C.	Acid-catalyzed hydrolysis method using 60 wt% H_2_SO_4_ at 45 °C for 1 h.	-	[77]

### 3.3. CNCs from Corncobs

Corn is a staple food in many countries, with about 1 billion tons of global annual production. In South Africa, approximately 16 million tons of corn were produced during the 2019/2020 period [78]. About 80% of the weight of the corn ear is attributed to corncob, and, together with the other components of the corn plant besides the corn kernels, are regarded as corn residue. Common uses of corncob residue include animal bedding, animal feed, and fertilizers. The majority of generated waste is burned in farms and/or landfills. Globally, various applications for corncobs are being developed, such as detergents, adsorbants, bioenergy feedstock, and composites. They are LCB waste with high cellulose content. Table 2 shows a few selected examples of CNC extraction processes using corncobs as a source. It is evident from the presented data that cellulose is present in varying amounts within the corn family and the isolation conditions contribute to the yield and physical properties (where CI* represents crystallinity index). Louis and Venkatachalam [79] demonstrated in their report that NaOH concentration, reaction time, and temperature all affect the yield of cellulose during the pre-treatment stage. The concentration of H_2_SO_4_ also affects the physical properties of CNCs, as shown in the table and in many reports in the literature. Recently, Adejumo et al. demonstrated the use of corncob, functionalised corncob, and CNCs as methyl orange dye adsorbants [80]. In their report, under optimum condition, CNCs had a calculated adsorption capacity of 206.67 mg/g, which was about 11.6 times greater than that of the corncob and 3.4 times higher than that of functionalized corncob [80].

### 3.4. Prospective Applications of CNCs

**Figure 5:**
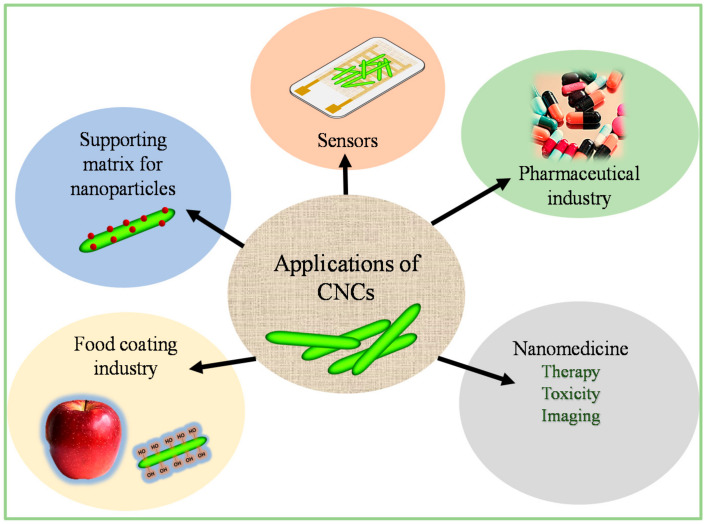
Different applications for CNCs reported in the literature.

Due to the abundance of biomass waste, various pre-treatment and extraction methods, outstanding and unique nanoscale structure, excellent mechanical properties, thermal stability, biocompatibility, biodegradability, and easy surface modification, CNCs have attracted rapidly growing scientific and technological interest, and have reported application prospects in many fields, such as health care, environmental protection, and chemical engineering [82]. Grishkewich et al. [83] summarized the recent applications of CNCs in biomedical engineering (tissue engineering, drug delivery, biosensors, and biocatalysts), wastewater treatment (adsorbents), energy, and electronics (supercapacitors, conductive films, substrates, sensors, and energy-storage separators). Figure 5 demonstrates the summarized recently reported applications of CNCs. CNCs have also found applications in the monitoring and improvement of food quality. Dhar et al. [84] fabricated a poly (3-hydroxybutyrate) (PHB)/CNCs based nanocomposite films with improved gas-barrier and migration properties for food packaging applications. Peng et al. [85] incorporated CNCs into different food-based systems containing polymers as a thickening agent. The CNCs improved the viscosity enhancement at lower particle loading. Besides these promising applications, CNC-based materials have also been applied in the fabrication of carbon-based nanomaterials. Dhar et al. used CNCs to prepare graphene with tuneable dimensions, while Souza et al. [86] prepared luminescent nanocarbon structures. Magagula et al. prepared luminescent-nitrogen-doped spherical carbons (NCSs) and used them for the detection of Fe^3+^ in aqueous solutions [77]. The following section focuses on the recent research regarding the fabrication of CQDs from LCB waste.

## 4. Carbon Quantum Dots (CQDs)

**Figure 6:**
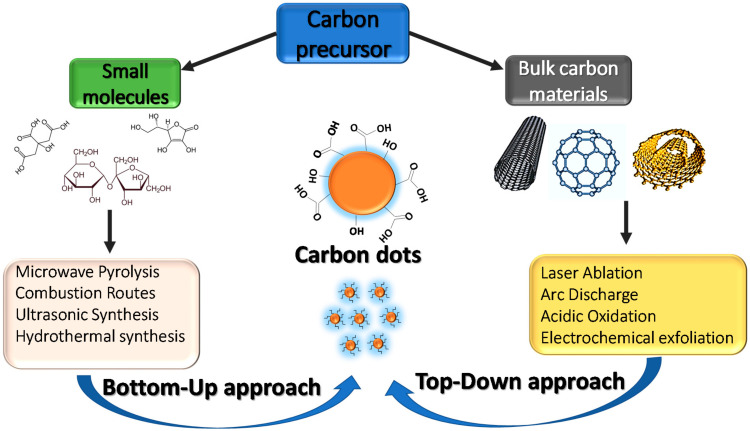
A schematic representation of the “top-down” and “bottom-up” approaches for the synthesis of CQDs.

**Figure 7:**
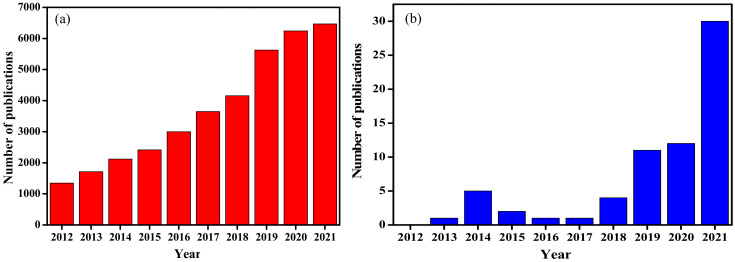
The number of publications containing “carbon dots” from 2012 to 2021 (**a**), and (**b**) the number of publications containing “carbon dots from agricultural waste” from 2012 to 2021. Data obtained from Web of Science in February 2022.

Over the past decade, extensive research has been conducted to explore synthesis methods for CQDs. These methods can be categorised into two main approaches known as the top-down and the bottom-up routes (Figure 6). The top-down method involves breaking down large carbon structures such as coal, activated carbon, graphite, and carbon nanotubes into the desired carbon nanostructures through electrochemical oxidation, acidic oxidation, arc discharge, and laser ablation [87]. The bottom-up route includes the polymerization and carbonization of small molecule precursors, such as citric acid, phenylenediamines, glucose, and aldehydes under a range of different reaction conditions through various chemical methods [88]. Some of these small-molecule precursors are found in varying amounts in biomass waste, making biomass waste an easily available and low-cost precursor. Despite the development of various fabrication strategies, the production of CQDs still requires complicated instrumentation, expensive precursors, and rigorous experimental conditions that present risks to the natural environment and human health. This further contributes to high production costs and commercialization constraints [89]. At present, the development of various fabrication strategies and mass production at low cost from renewable and green sources is of great interest. Figure 7a shows the number of publications with the phrase “carbon dots” for the past decade. From just over one thousand in 2012 to over six thousand in 2021, there is clearly a growing interest in research related to these materials.

LCB waste presents carbon (C) sources that are rich in elements such as nitrogen (N), hydrogen (H), and oxygen (O), in addition to C. These sources are renewable, cost-effective, and environmentally benign compared to other carbon sources. The production of CQDs from LCB waste converts low-value biomass waste into valuable and useful materials. Zhou et al. [90] proposed a green synthesis method by utilizing watermelon peel as the carbon precursor for the first time, starting a new trend towards using biomass waste materials for CQD preparation. Following this, researchers have utilized different types of agricultural waste, animal waste, fruit waste and vegetable waste. Figure 7b shows a publication trend for the past decade where the phrase “carbon dots from agricultural waste” was used. There is a clear jump in the amount of research done in this topic in 2021, which is more than twice as much as the number of appearances in previous years. This sudden increase is in line with current government policies on waste management across the world as well as the United Nation’s 2030 sustainable development goals. 

Due to differences in biomass composition, CQDs derived from different agricultural residues and synthesis techniques show different luminescent properties, size distributions, and quantum yields (QYs) [91]. Conventional methods involved in preparation of agricultural waste-based CQDs require complicated equipment, catalysts, several post synthesis purification steps, longer synthesis times, and harsh experimental conditions, which results in expensive production costs [12]. Therefore, exploration of green synthesis methods with fewer synthesis steps, minimum use of toxic chemicals, and reduced synthesis time is necessary. At present, microwave-assisted synthesis is highly desirable due to its simplicity, short synthesis time, low cost, and homogeneous heating [92]. In our recent study, spherical carbons (CSs) were successfully fabricated from corncob via alkaline treatment, acid hydrolysis, and microwave synthesis, and were subsequently applied in the fluorescent detection of Fe^3+^ in aqueous solution [77].

### 4.1. Properties of CQDs

CQDs are the most-desired alternative to toxic, heavy-metal based QDs for fluorescence-related applications due to their high fluorescence stability, environmental friendliness, good biocompatibility, facile synthesis, and low toxicity [10]. These properties strongly depend on several factors, including synthesis technique, chosen precursors, post-synthesis treatments, time and temperature of the synthesis, pH, surface passivation or functionalization, heteroatom doping, and so on [13]. Not only do these factors affect the microstructure of CQDs, but also the optical properties, and QY. In the following section, the physical, chemical, and optical properties of CQDs are discussed in greater detail. 

#### 4.1.1. Structural Properties

CQDs are typically nanoparticles smaller than 10 nm, composed of a core–shell structure with sp^2^/sp^3^ carbon cores functionalized with polar oxygen groups [11]. The existence of the surface groups of CQDs depends mainly on the type of precursor used in the synthesis. When the used precursor is heteroatom-rich, the surface tends to have modified-functional groups such as carboxyl, amine, carbonyl, and ether groups [93]. The surface functional groups impart CQDs with excellent water solubility and also ease further surface functionalization with various molecules [94]. In addition, the precursor and synthesis methods also determine the composition, morphology, and size distribution of the synthesized CQDs. Various characterization techniques are applied to determine the physical properties and the crystalline structure of the CQDs. These techniques include atomic force microscopy (AFM), high-resolution transmission electron microscopy (HRTEM), X-ray diffraction (XRD), and Raman spectroscopy [10]. To investigate their chemical structure, X-ray photoelectron spectroscopy (XPS), element analysis, Fourier transform infrared (FTIR), and nuclear magnetic resonance (NMR) are used [95]. Characterization of CQDs is essential for attaining a better understanding of the mechanisms associated with the unique structural properties of the CQDs. 

The morphology and size distribution of CQDs can be measured from TEM images, while AFM is used to measure the height information of CQDs. At present, most biomass-based CQDs are usually spherical, with the average particle size less than 10 nm in a state of uniform dispersion [96]. Smaller-sized CQDs have been obtained from eggshell membrane peel [97], pomelo peel [98], and garlic husk [99]. CQDs with larger size distributions have also been obtained from spent tea, with an average size distribution of 11 ± 2.4 nm [89], and from goose feathers with an average size distribution of 21 ± 5 nm [100]. The crystalline properties are determined using HRTEM, XRD, and Raman spectroscopy. HRTEM is used to determine the lattice fringe spacing of the carbon materials, which largely corresponds to the different diffraction planes. Atchudan et al. reported HRTEM imaging of CQDs prepared from banana-peel waste with a lattice spacing of 0.21 nm [101]. The XRD pattern of CQDs generally presents a broad diffraction peak between 2θ values of 20° to 25° and lattice spacing between 0.31 and 0.38 nm [102]. An AFM image of pine-wood-based CQDs with an average height of 2.8 nm, corresponding to 5–7 layers of graphene, was reported by Zhao et al. via a 3D morphology presentation [103]. 

The graphitization/crystallization of CQDs is examined by Raman spectroscopy. Raman spectra of CQDs exhibit two broad peaks at around 1300 cm^−1^ and 1580 cm^−1^ (similar to other graphene-derived carbon materials), which are attributed to the D (sp^3^-hybridized) and G (sp^2^-hybridized) bands, respectively [104]. The D band is associated with the vibrations of carbon atoms with dangling bonds in the termination plane of disordered graphite, and the G-band is related to the in-plane vibrations of sp^2^-hybridized carbon. Hence, the intensity ratio of D to G (I_D_/I_G_) is the measure of the defects present on the graphitic structure; a low I_D_/I_G_ ratio represents that the integrity of the graphitic shells is sufficiently high to protect the core material well from corrosion and oxidation [105]. The surface functional groups and elemental composition of CQDs are examined by FTIR and XPS. FTIR spectroscopy is used to understand the surface functional groups contained on the CQDs. CQDs usually exhibit main characteristic absorption bands of O–H, C–H, C=C, and (C=O). Other LCB-waste-based CQDs may contain nitrogen and sulphur depending on the chemical composition of the precursor [106]. XPS analysis is carried out to delineate the chemical composition and nature of bonding in CQDs. LCB-waste-based CQDs generally contain carbon, oxygen, nitrogen, and sulfur, which can be detected in XPS. XPS can be used to determine the elemental composition and the chemical and electronic states of the contained elements. CQDs usually show three apparent peaks centred around 283, 400, and 530 eV, which are attributed to C_1s_, N_1s_, and O_1s_, respectively [107].

#### 4.1.2. Optical Properties

Due to quantum-confinement effect, the optical properties are the most notable properties of CQDs, irrespective of their microstructure. CQDs possess excellent optical properties, such as wavelength-tuned emissions, which may be affected by surface states, surface passivation, heteroatom doping, and surface defects. This section presents and discusses the optical properties of CQDs.

##### UV-Absorption Properties

**Figure 8:**
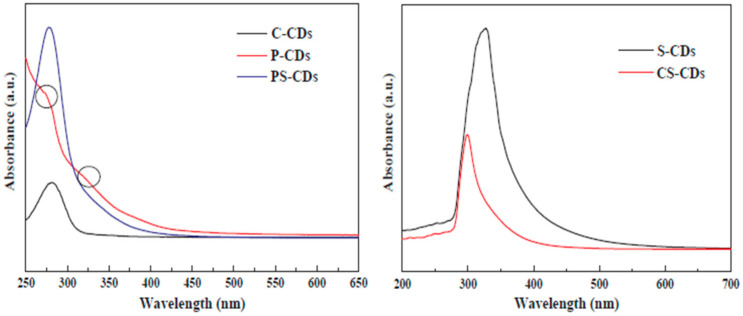
UV–vis spectra of (**left**) C-CDs, P-CDs, and PS-CDs and (**right**) CS-CDs and S-CDs. Adapted with permission from [108], Copyright {2020}, Elsevier.

CQDs usually show a strong absorption peak in the UV region and a lower absorption peak in the visible region [102]. UV absorption (around 230–270 nm) is induced by the π–π* transition of C=C and C=N bonds, while visible absorption (around 300–330 nm) is ascribed to n–π* transition of C=C or C=O bonds [91]. The UV–vis spectra of dwarf-banana-peel CQDs showed two absorption peaks appearing at 272 nm and 320 nm [101]. These were attributed to the π–π* transition of C=C bonds and the n–π* transition of C=O bonds in CQDs, respectively [101]. Moreover, the nature of the CQD precursor and surface functional groups can affect the position and intensity of the absorption peaks. Liu et al. [108] prepared CQDs from different agricultural waste materials (cellulose-based CDs (C-CDs), protein-based CDs (P-CDs), peanut shell-based CDs (PS-CDs), cotton stalk-based CDs (CS-CDs), and soymeal-based CDs (S-CDs)). As shown in Figure 8, two absorption peaks at 273 nm and 322 nm were observed for the P-CDs, while only one absorption peak was observed for the rest of the samples (at 281 nm for C-CDs, 278 nm for PS-CDs, 299 nm for CS-CDs, and 328 nm for S-CDs) [108].

##### Fluorescence Properties

**Figure 9:**
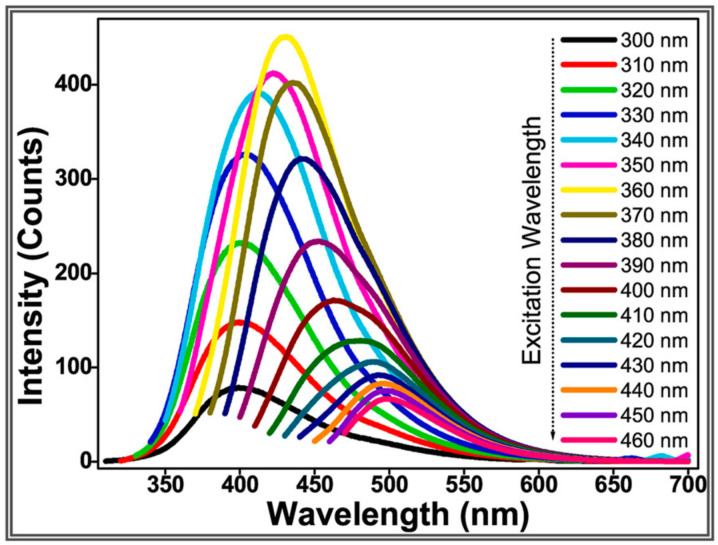
Fluorescence emission spectra at different excitation wavelengths of CQDs prepared from kiwifruit peels. Adapted with permission from [111], Copyright {2022}, Elsevier.

The fluorescence properties of CQDs are quite fascinating and can affect the application of CQDs in different fields. CQDs possess excellent fluorescence properties, including excitation-wavelength-dependent fluorescence, size-dependent fluorescence emission, up-conversion luminescence, strong resistance to photobleaching, and good fluorescence stability [109]. The photoluminescence (PL) emission of CQDs occurs when trap states are present in the bandgap (caused by impurities, surface defects, functional groups, and adsorbed molecules). In such cases, the photoexcited electron or hole can be trapped, and the following recombination leads to a radiative emission of energy [110]. The observed CQD PL can be due to the combination of different mechanisms from different sources, the surface state, quantum confinement effect, and molecular state mechanisms [102]. Most of the CQDs reported so far have a common feature of presenting excitation-dependent emission, giving a decrease in the emission signal that is systematically displaced towards longer wavelengths as the excitation wavelength is increased [94,111]. Atchudan et al. reported an excitation-wavelength-dependent fluorescence emission of CQDs that were prepared from kiwifruit-peel waste (Figure 9) [111]. The intensity peak of the CQDs initially increased from 300 to 360 nm excitation wavelength but decreased from 360 to 460 nm excitation wavelength. A fluorescence-emission red-shift was also observed with increasing excitation wavelength [111]. However, excitation-independent emission of biomass-waste-based CQDs was recently reported by Abbas et al. [89].

Heteroatom doping, surface functionalization, and surface passivation of CQDs are known to induce surface-state mechanisms. Heteroatom doping is a common method in the preparation of CQDs and allows their intrinsic properties to be tuneable and exploited for their desired potential applications. Elements such as N, B, S, and P are used as a dopants to replace carbon atoms in the sp^2^/sp^3^ network [109]. Surface functionalization is related to the introduction of functional groups via covalent bonding on the carbon edge planes [112]. Surface passivation involves the coating of passivating reagents such as polyethylene glycol (PEG), amine terminated polyethylene glycols (PEG-1500N), poly(ethylenimide)-co-poly(ethyleneglycol)-co-poly(ethyl-enimide) (PPEI), 4,7,10-trioxa-1,13-tridecanediamine (TTDDA), and polyethyleneimine (PEI) on the surface of the carbon core of CQDs to regulate their surface state [113]. In general, the surface states of CQDs produce a variety of energy levels and lead to various emissive traps [102]. Monday et al. [114] prepared nitrogen-doped CQDs (N-CQDs) from palm-kernel shells using ethylenediamine and L-phenylalanine as dopants. The as-prepared N-CQDs showed fascinating PL properties, with a QY of 13.7% for ethylenediamine doped N-CQDs and 8.6% for L-phenylalanine doped N-CQDs, as well as an excitation-dependent emission wavelength [114]. Chen et al. [115] prepared N, S co-doped NQDs from used garlic, which displayed strong fluorescence with a QY of 13%. N, P co-doped CQDs with a QY as high as 76.5% were synthesized by Dong and colleagues [116]. From the few literature sources that have been quoted here, it is quite clear that uniformity in these types of material does not exist. This justifies the extensive research that is carried out in order to qualify a set of CQDs to a specific application. Further, the differences in the properties of CQDs make them interesting for a wide range of applications. 

### 4.2. Potential Applications of CQDs

**Figure 10:**
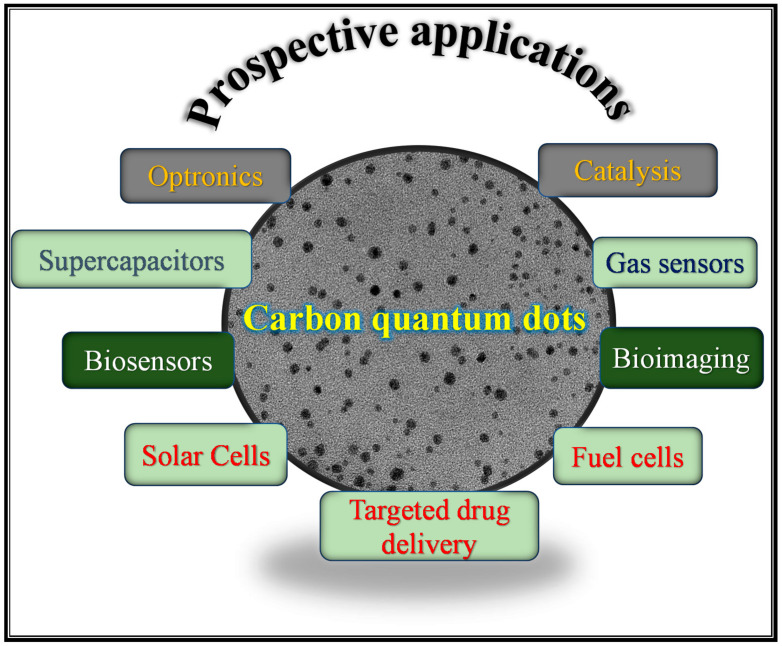
Prospective application for carbon quantum dots.

**Figure 11:**
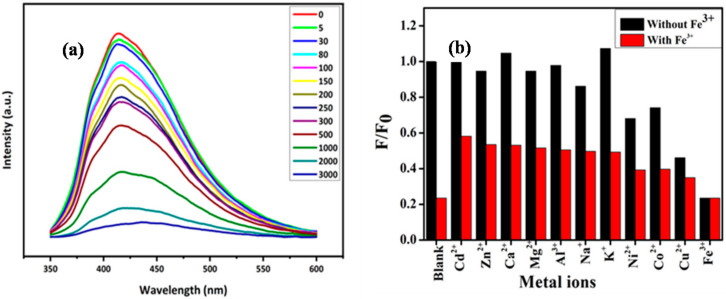
(**a**) Fluorescence spectra of NCSs with different concentrations of Fe^3+^ (5–3000 μM); (**b**) Changes in the fluorescence intensity ratio (F/F_0_) of N-CSs after the addition of various metal ions. Adapted with permission from [77], Copyright {2021}, IEEE.

CQDs have promising application in bioimaging, biosensing, fuel cells, supercapacitors, catalysis, solar cells, lithium-ion batteries, drug delivery, and light-emitting diodes due to their outstanding chemical, physical, and optical properties (Figure 10) [13]. According to a Web of Science search done by Li et al. [109] in March 2021, 41% of all published papers on CQDs reported their potential application in sensors (the only application discussed in this section). This is due to their strong luminescence properties and sensitivities towards specific metal ions in aqueous environments. CQD-based sensors give rise to a low limit-of-detection (LOD) and high sensitivity and selectivity [117]. Wang et al. [118] proposed a CQD-based PL sensor for the first time and demonstrated that the luminescence of CQDs can be quenched selectively by Fe^3+^ through a charge-transfer mechanism, starting a new trend towards using CQDs for the detection of heavy-metal ions. Zhao et al. [119] prepared water-soluble, luminescent N-CQDs from chitosan and utilized them for the sensing of Fe^3+^ in aqueous solutions. The N-CQDs presented outstanding selectivity and sensitivity and were successfully applied for the quantitative detection of Fe^3+^ with a linear detection range of 0–500 μM and an LOD of 0.15 μM. Magagula et al. reported corncob-derived NCSs (which contained a high concentration of CQDs) for the detection of Fe^3+^ in aqueous solution [77]. In their report, a linear detection range of 0–500 μM and an LOD of 70 nM was reported for Fe^3+^. The quenching effect of Fe^3+^ on NCSs was demonstrated through a gradual increase of Fe^3+^ concentration from 5 µM to 3000 µM (Figure 11a). Furthermore, different metal ions were selected to demonstrate the selectivity of NCSs to Fe^3+^, as shown in Figure 11b [77]. N, P co-doped CQDs were adopted as a fluorescent sensor for the effective detection of Fe^3+^ in water, with an LOD of 0.1 μM, and the sensor showed a better linear relationship in the range of 0.1∼50 μM [116]. High-luminescence S-CQDs were synthesized from cellulose fibres with a QY of 32%, and these were utilized to detect Fe^3+^ in pH 0 solutions and showed excellent selectivity and sensitivity with an LOD of 0.96 μM [120]. Table 3 shows selected articles listing the synthesis conditions, some properties, and reported potential applications of CQDs obtained from biomass waste precursors. This table confirms the versatility of CQDs as well as their inconsistent quantum yield. In addition to Fe^3+^, CQDs can be applied in the sensing of various transition-metal ions such as Hg^2+^, Cu^2+^, and Pb^2+^. Moreover, CQDs have also been applied in other sensing systems such as biosensing and chemical sensing; however, PL sensing is currently the most-reported potential application for these materials. 

## 5. Conclusions and Outlook

In recent years, LCB waste has been utilized to prepare CNCs with different attributes and applications. This choice of a feedstock is renewable, green, and affordable. Several modifications of the pre-treatment and extraction stages continue to be explored, with the aim of attaining CNCs with desired attributes (including their production at a commercial scale). In addition to the applications of CNCs in pharmaceuticals, medicine, composite materials, energy, and packaging, their application as a precursor in synthesis of carbon nanomaterials is gaining momentum. This is in line with developing new greener methods of material synthesis as well as finding cheaper ways of producing smart materials. CQDs seem to be much easier to fabricate from LCB waste sources when compared to other types of carbon nanomaterials. Several advantages and disadvantages exist in both the fields of CNC extraction and CQD fabrication from LCB waste. The popular chemical treatment method of LCB in order to obtain CNCs is lengthy and requires the continuous use of strong acids in order to completely separate the amorphous content from the cellulose. This is not environmentally friendly. The use of strong acids and bases also implies that large amounts of water will be used to purify the CNCs. Compared to other extraction methods, chemical methods are cheaper; however, process efficiencies should be evaluated.

The application of LCB waste in the fabrication of carbon nanomaterials is a promising field with the potential to transform the agricultural sector as we know it. CQDs are versatile materials with the potential to replace many toxic, heavy-metal-based optoelectronic devices. Based on the current research trends, we can predict that CQDs will be popular in the market in the near future. However, large variations in the properties of CQDs as the result of differences in feedstocks means that CQDs can be optimised for a specific application per batch. Further studies on large-scale synthesis of CQDs are yet to be explored.

## Figures and Tables

**Table 2:** Selected examples presenting the extraction of CNCs from corncob residue.

Source	Components	Isolation Conditions	Yield (%)	CI* (%)	Average Size (nm)	References
**Corncob**	Cellulose 34.11 ± 1.47%, lignin 15.08 ± 1.32%, hemicellulose 20.17 ± 2.43%, ash 30.06 ± 1.36%, other components 0.58 ± 0.11%.	Ball milling machine for 2 h in DMSO	41.21	70.58	174.27 ± 4.32	[81]
**Corncob**	Cellulose 45.01 ± 0.9%, hemicellulose33.12 ± 1.1%, lignin 13.81 ± 1.3%, ash 3.1 ± 0.5, other extractives 4.96 ± 1.1%.	60% H_2_SO_4_ at 45 °C for 1 h.	-	72.36	131.4	[79]
**Corncob**	Cellulose 63.5%, xylan 2.7%, lignin 25.8%, ash 2.1%.	64% H_2_SO_4_ at 45 °C for 1 h	34.5	55.9	198 ± 51	[38]
**Corncob**	Cellulose 63.5%, xylan 2.7%, lignin 25.8%, ash 2.1%.	0.5% HCl and 88% CH_2_O_2_ at 95 °C for 30 min.	66.3	63.8	421 ± 112	[38]
**Corncob**	Cellulose 63.5%, xylan 2.7%, lignin 25.8%, ash 2.1%.	TEMPO (1 mmol/L) and sodium bromide (10 mmol/L) at pH 10.	78.4	49.9	438 ± 173	[38]

**Table 3:** Selected articles showing the fabrication, properties, and applications of CQDs using biomass waste as a precursor.

Source	Synthesis Method and Conditions	Fluorescence Quantum Yield	Application	References
**Watermelon peels**	Pyrolysis 220 °C, 2 h	-	Cell imaging	[90]
**Orange peels**	Hydrothermal 180 °C	-	Photocatalysis	[121]
**Sago waste**	Pyrolysis 250 °C to 450 °C, 1 h	-	Detection of Cu^2+^ and Pb^2+^	[12]
**Wheat straw**	Hydrothermal 250 °C, 10 h	20%	Biomedical labelling, imaging, and detection of Fe^3+^	[122]
**Prawn shells**	Hydrothermal 180 °C, 8 h	9%	Detection of Cu^2+^	[123]
**Peanut shells**	Pyrolysis 400 °C, 4 h	10.58%	Detection Cu^2+^	[124]
**Expired milk**	Hydrothermal 180 °C, 2 h	-	Detection of Fe^3+^, bioimaging, preparation of FCDs/SiO_2_ nanocomposites, and fluorescent ink for patterning.	[125]
**Rice husk**	Hydrothermal 200 °C, 6 h	3%	Detection of alcohol vapours at room temperature.	[126]
**Palm-kernel shell**	Microwave 385 W, 1 to 5 min	44.0%	Bioimaging, detection of Cu^2+^, and removal of heavy-metal ions (Cu^2+^).	[127]
**Corncob-derived CNCs**	Microwave 180 °C, 10 min	-	Detection of Fe^3+^	[77]

## Data Availability

Not applicable.
